# A Clinico-Histopathological Study of Lupus Vulgaris at a Tertiary Care Centre

**DOI:** 10.7759/cureus.42710

**Published:** 2023-07-30

**Authors:** Binodini Behera, Sonal Jain, Liza Mohapatra, Vaishali Masatkar, Sibaram Panda

**Affiliations:** 1 Department of Dermatology, Pandit Raghunath Murmu Medical College, Baripada, IND; 2 Department of Dermatosurgery and Hair Transplantation, CUTIS Hospital, Bengaluru, IND; 3 Department of Dermatology, Institute of Medical Sciences and SUM Hospital, Bhubaneswar, IND; 4 Department of Dermatopathology, NIMS Hospital, Jaipur, IND

**Keywords:** tuberculosis luposa, skin tuberculosis, papulosquamous, lupus vulgaris, cutaneous tuberculosis

## Abstract

Background and objectives

Lupus vulgaris is a chronic, progressive, paucibacillary form of cutaneous tuberculosis that occurs in persons with moderate to high immunity. Due to its varied clinical presentation, it can masquerade as different dermatological conditions. This study describes the demographic patterns and varieties of clinical manifestations that can be possible in this curable illness.

Methods

This study was conducted over two years and included 19 patients with histopathologically confirmed lupus vulgaris in Odisha, India. Demographic data, clinical features, and response to treatment are presented.

Results

Thirteen cases (68.4%) were seen in adults and six (31.6%) in pediatric patients. The lower limbs were the more affected (n=10), followed by the upper limb (6), the face (2), and the chest (1). All but one patient had plaque-type lesions. On histopathology, all showed a tuberculoid granuloma with no demonstration of acid-fast bacilli with Ziehl-Neelsen staining.

Conclusion

In the present study, the incidence was mostly observed in the young and higher activity age groups (5-40 years). Plaque-type lesions were most commonly encountered. In histopathology, all the cases had tubercular granuloma-type lesions without any incidence of malignant transformations. All the patients responded well to conventional multi-drug anti-tubercular chemotherapeutic regimens.

## Introduction

Tuberculosis (TB) is one of the oldest known diseases and still continues to be a burden in certain parts of the globe, frequently drawing attention to medical professionals and society as a whole. Cutaneous TB is an important form of tuberculosis, with an incidence of about 5.9 cases per 1,000 people [1]. It is caused by *Mycobacterium tuberculosis* complex, *Mycobacterium bovis*, and sometimes the Bacillus Calmette-Guérin (BCG) vaccine as well. It presents in varied clinical forms depending on the immunity status of the person, environmental influence, and type of exposure [2]. Lupus vulgaris (LV) is the most common form of cutaneous TB, accounting for 75% of the cases. LV is a chronic, progressive, post-primary, paucibacillary form of cutaneous tuberculosis occurring in persons with a moderate or high degree of immunity [3]. It can develop as a result of direct inoculation, extension from an underlying organ, lymphatic spread, or rarely, hematogenous spread from an infective focus. Due to its varied clinical presentation, LV can mimic many dermatological conditions. The most important histopathologic feature is the formation of tuberculoid granulomas with or without caseation, surrounded by epithelioid histiocytes and multinucleated giant cells. Secondary changes such as atrophy, thinning, hypertrophy, or hyperkeratosis have the potential to influence clinical presentation. Sometimes, pseudo-epitheliomatous hyperplasia may also be noted. Usually, acid-fast bacilli (AFB) are absent, as most of the lesions are paucibacillary [4]. In the present series, we aim to describe the possibilities of different clinical and demographic patterns of LV with histopathological confirmation and observe their response to anti-tubercular therapy.

## Materials and methods

This study was conducted in the Department of Dermatology and Venereology of Pandit Raghunath Murmu Medical College, Baripada, Odisha, India, over a period of two years starting from April 1, 2020, to March 31, 2022.

Patients with clinically suspected cases of LV are admitted to the dermatology ward of our hospital. The demographic profile, including age, sex, and profession, along with a comprehensive medical history, clinical examination findings, complete hemogram with erythrocyte sedimentation (ESR) and C-reactive protein (CRP), liver function test, Mantoux test, and chest X-ray, are documented in the patient's case sheet. Additionally, patients undergo enzyme-linked immunosorbent assay (ELISA) screening for HIV. To confirm the diagnosis, microbiological studies (cartridge-based nucleic acid amplification testing (CBNAAT) and AFB tests) are conducted using tissue exudate, along with histopathological analysis through punch biopsy.

Among the 85 clinically suspected cases of admitted patients in the study period, 19 patients with positive histopathology results were included in the study. These patients were initiated on a six-month regimen of oral multi-drug anti-tubercular chemotherapy. The initial two-month phase involved a four-drug regimen comprising Isoniazid (INH), rifampicin, pyrazinamide, and ethambutol, followed by a four-month phase of rifampicin and INH. Subsequent follow-up assessments of the patients were conducted by two authors from the Institute where the study took place. Additionally, some patients were prospectively monitored from the beginning of the study to observe the healing status of the lesion.

## Results

A total of 19 cases of LV were reported during the study period. The female-to-male ratio was 1.1:1 . Fourteen (73.7%) cases occurred in young patients in the age range of 5-40 years. Dividing by careers, it was seen that six students were affected, followed by four housewives, three farmers, three daily wage workers, two drivers, and one industry worker. Eighteen patients (94.73%) presented with plaque-type LV; the only patient with vegetating type was a child.

Among the patients with plaque-type LV, three patients had multifocal disease. A history of pulmonary tuberculosis was positive in three out of 19 patients. None of the cases had a family history of TB. Eight (42.10%) patients had a history of trauma. The lower limbs, including the gluteal region, were the most common site involved (10 patients), followed by the upper limbs in six, the face in two, and the chest in one patient. The age and occupation, site of involvement, and type of lesion are shown in detail in Table 1. Positive Mantoux test was seen in five (26.3%) cases.

**Table 1:** Demographic details of the cases

S. no	Age (in years)	Sex	Profession	History of truma	Site	Type of lesion
1	52	M	Farmer	Present	Chest	Plaque
2	21	F	Student	Absent	Upper limb (Elbow)	Plaque
3	9	M	Student	Absent	Gluteal area	Plaque
4	18	F	Industry worker	Present	Lower limb	Plaque
5	59	M	Farmer	Present	Lower limb	Plaque
6	10	F	Student	Absent	Face(perioral and perinasal area)	Vegetating
7	68	F	Housewife	Absent	Lower limb	Plaque
8	38	M	Driver	Absent	Upper limb (Antecubital fossa)	Plaque
9	5	M	Student	Absent	Gluteal area	Plaque
10	34	F	Housewife	Absent	Lower limb	Plaque
11	55	F	Housewife	Present	Lower limb	Plaque
12	75	F	Housewife	Absent	Upper limb (Antecubital fossa)	Plaque
13	9	M	Daily wage worker	Present	Lower limb	Plaque
14	8	M	Student	Absent	Upper limb (Elbow)	Plaque
15	36	M	Driver	Present	Upper limb(Right index finger)	Plaque
16	7	F	Student	Absent	Gluteal area	Plaque
17	27	F	Daily wage worker	Present	Upper limb (Right thumb)	Plaque
18	34	M	Farmer	Absent	Lower limb	Plaque
19	25	F	Daily wage worker	Present	Face	Plaque

**Figure 1:**
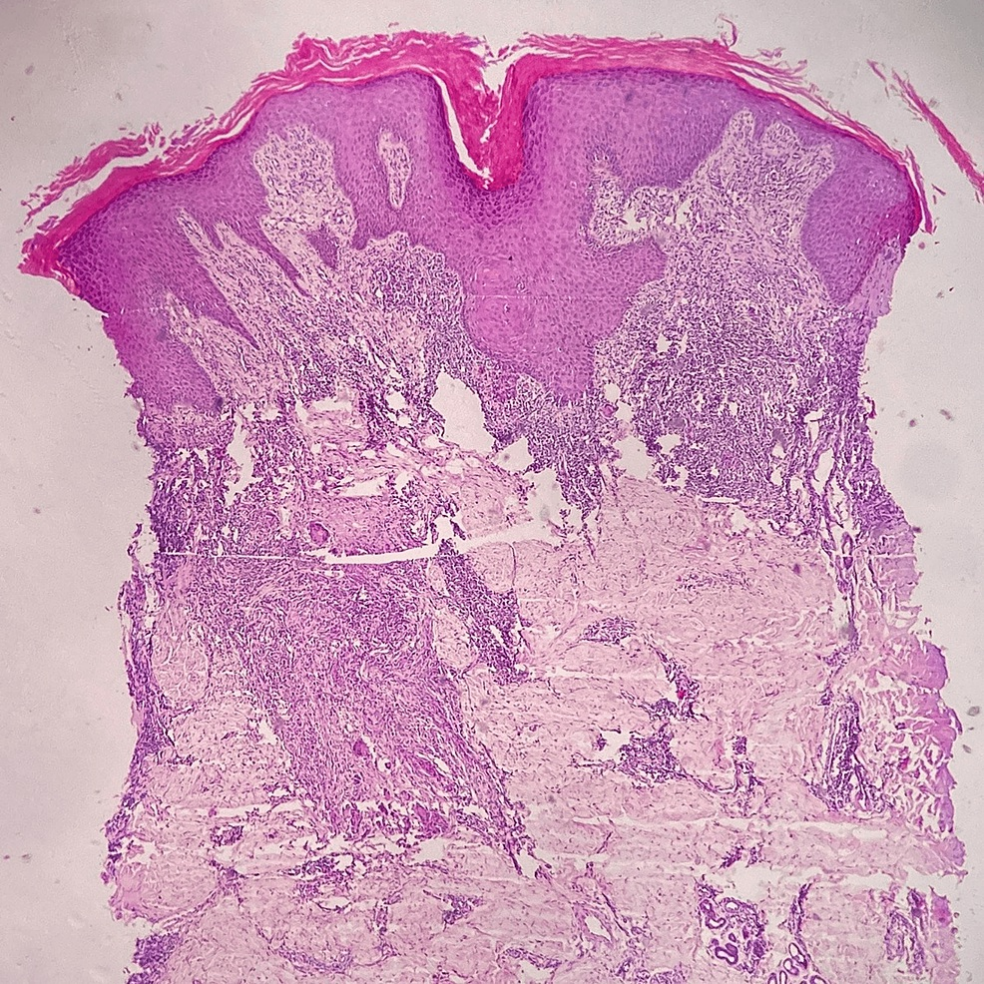
Histopathological (H&E) image (4x) showing hyperkeratosis, follicular plugging, hypergranulosis, irregular acanthosis with pseudoepitheliomatous hyperplasia, diffuse chronic inflammatory infiltrate in upper dermis with well-formed non-caseating granulomas in mid-dermis.

**Figure 2:**
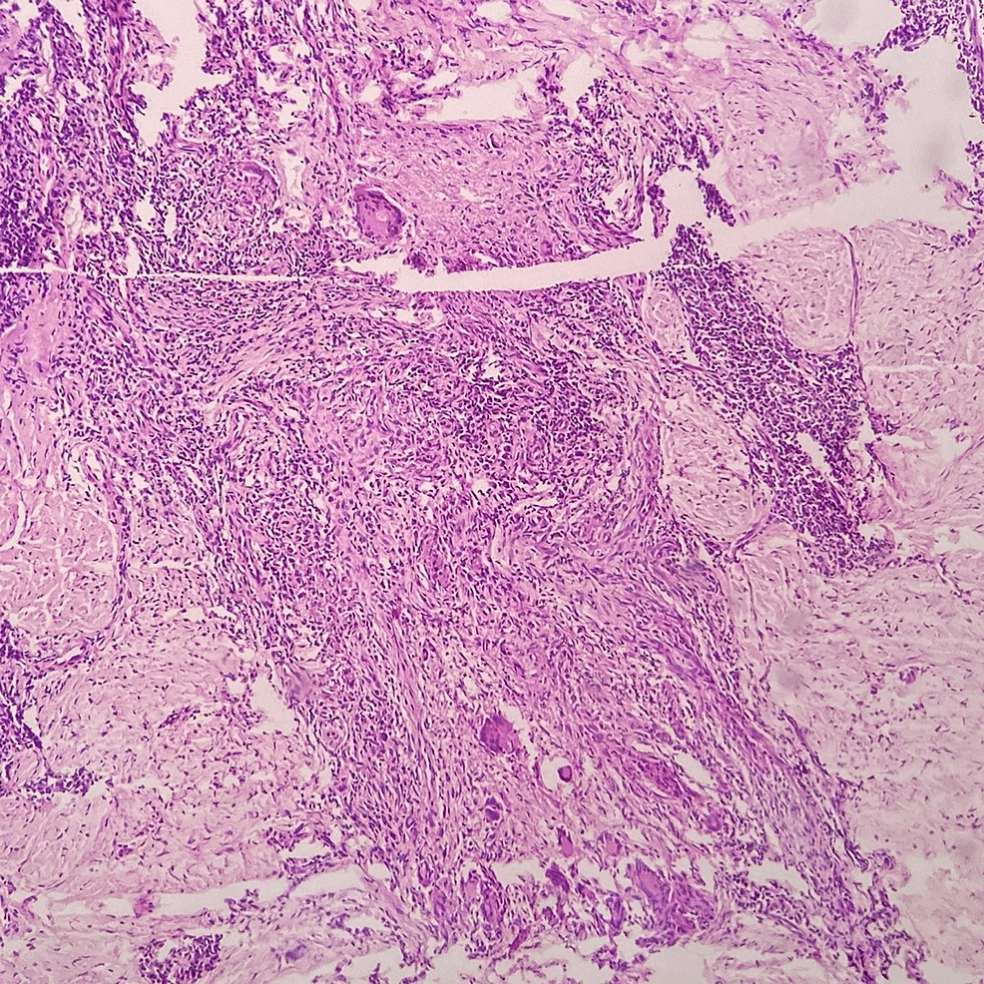
Histopathological (H&E) image (10x) showing dense infiltrate comprising of lymphocytes, histiocytes and multi-nucleated Langhans-type giant cells in mid dermis.

Histopathological examination was performed in all the cases, which demonstrated epidermal hypertrophy in 15 patients, and among these, 10 had pseudoepitheliomatous hyperplasia (Figure 1), while four had epidermal atrophy. Well-circumscribed collection of epithelioid histiocytes with surrounding dense infiltrate of lymphocytes was noticed in all the cases, predominantly in the upper and mid dermis (Figure 2). Sixteen cases showed multi-nucleated Langhans-type giant cells. Acute inflammatory infiltrate including neutrophils and eosinophils were present in three cases. Caseous necrosis was absent in all the cases. None of the cases demonstrated AFB on Ziehl-Neelsen staining. Periodic acid Schiff (PAS) stain was also performed in all the cases to rule out deep fungal infection but none of them were positive.

## Discussion

LV or the “wolf” form of TB is the most common form of cutaneous TB in adults in India [5]. LV accounts for 59% of cutaneous TB cases in India [6]. Cutaneous involvement in TB cases worldwide is observed in approximately 1-2% of all affected individuals and is commonly encountered, particularly in developing and tropical regions [7]. The overall prevalence of cutaneous  TB in various Indian studies is 0.25-0.6% [8]. LV is a post-primary paucibacillary cutaneous TB that occurs in previously sensitized individuals with a moderate degree of immunity. It is usually a re-infection of TB of the skin and this is acquired either exogenously by direct inoculation of the bacilli or endogenously by hematogenous or lymphatic spread from an underlying infected focus. Sometimes, it may develop exogenously following inoculation for tuberculosis secondary to Bacille-Calmette-Guerin vaccination [9].

Childhood TB represents 5-15% of all cases of TB [10]. In our study, six (31.6%) patients belonged to the pediatric age group. Fourteen patients (73.68%) were seen in the age group of 5-40 years, probably due to their higher activity level and their being more prone to injury.

It is characterized by brownish-red papules or nodules that gradually extend peripherally with healing and scarring of initial lesions. It can present in different clinical patterns which include papular, nodular, plaque, ulcerative, vegetative, hypertrophic, atrophic, tumor-like, and mutilating types [11]. The ulcerative and mutilating variants of LV can cause scarring, ulceration, and crusting over areas of necrosis with the destruction of deep tissue and cartilage resulting in deformity while vegetating forms can produce marked infiltration, ulceration, and necrosis with minimal scarring [12]. All but one patient in this study had plaque-type LV. No ulcerative, tumor-like, or papulonodular forms of LV were observed. The diverse manifestations of LV can be attributed to multiple factors, including the patient's age, disease duration, body site affected, vascularity of the area, mode of entry, patient immunity, and antigenic load.

LV exhibits a higher prevalence in the facial region in Western countries, owing to its relatively cooler climate compared to other areas. Reports indicate that approximately 80% of lesions are observed in the head and neck, specifically in the nose and cheeks, in European countries, and approximately 62% of lesions were found on the face in Türkiye [13,14]. In India, the buttocks and lower limbs are more frequently affected by recurrent pyoderma and trauma due to compromised skin integrity.

Histopathological examination of LV may reveal either atrophic or hypertrophic epidermis characterized by acanthosis, papillomatosis, and occasionally pseudo-epitheliomatous hyperplasia or ulceration. Tuberculoid granulomas comprising lymphocytes, plasma cells, epithelioid cells, and giant cells, are observed in the superficial dermis, with limited or absent central caseation. The demonstration of tubercle bacilli is challenging [15,16]. Mycobacterial DNA detection tests using polymerase chain reaction (PCR) are more sensitive but their interpretation is also difficult [17]. We have not used this method in our study.

The histopathological differential diagnosis of LV includes sarcoidosis, tuberculoid leprosy, granulomatous foreign body reactions, and deep fungal infections. In tuberculoid leprosy, the granulomas are found predominantly around dermal nerves and appendages. Sarcoidosis shows naked granulomas whereas granulomatous foreign body reaction reveals the agent and deep fungal infections demonstrate fungal elements in PAS stain [18]. Sixteen cases (84.21%) in the present study revealed tuberculoid granuloma compared to previous studies where they have found in 70% of cases [19,20].

The culture for TB bacilli in LV frequently comes out to be negative and only a 6% positivity rate has been reported in the literature [21]. Similarly, we did not find ACB in culture or tissue.

Malignant tumors are known to occur in LV and have a reported rate of 0.5-10.5%; squamous cell carcinoma being the most common form [22]. However, the follow-up was not long enough to determine this statement.

**Figure 3:**
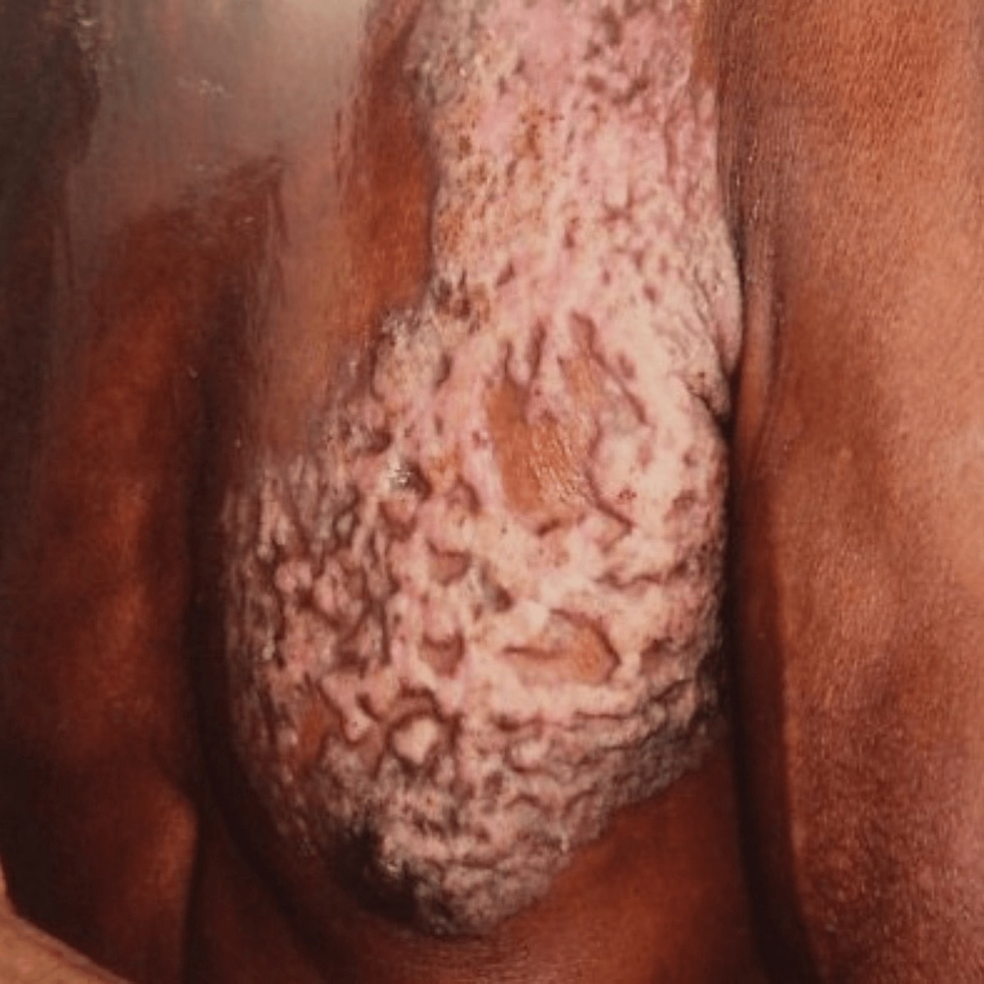
Cribriform plaque-type lesion, before treatment.

**Figure 4:**
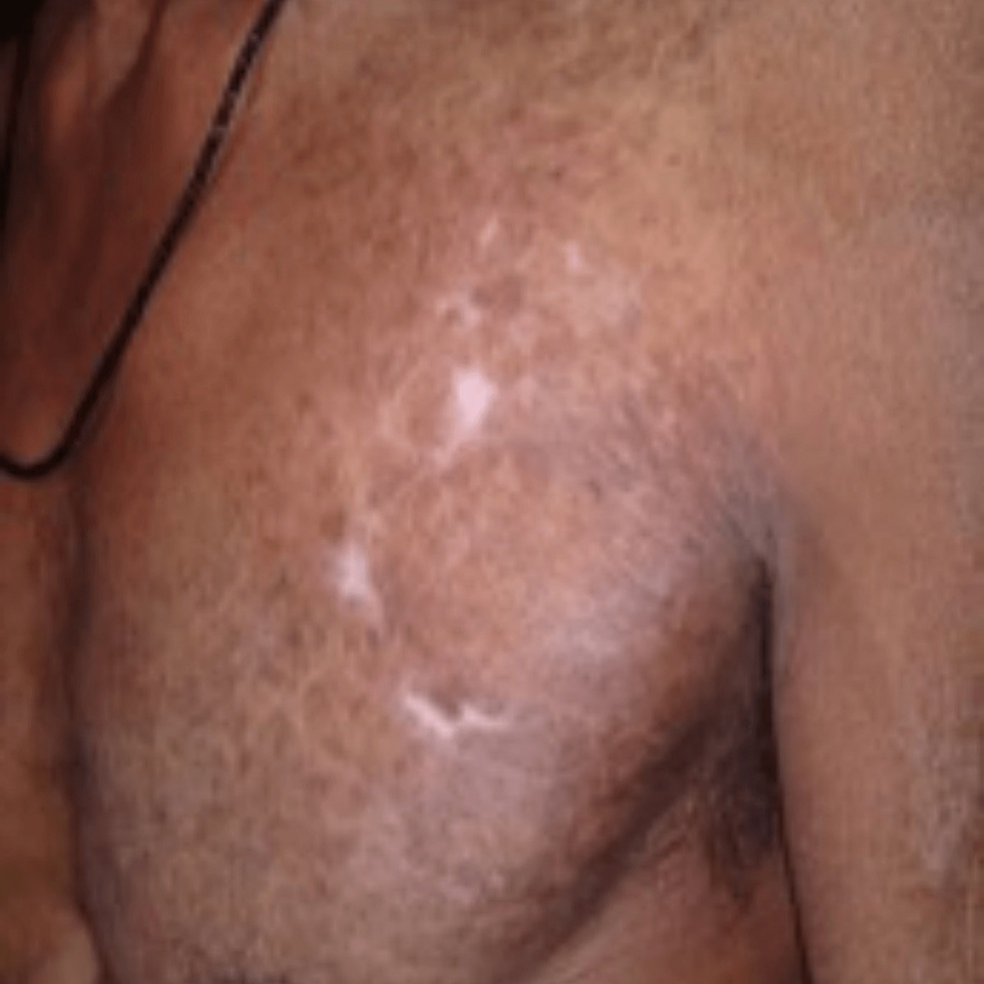
Healed cribriform plaque-type lesion, after treatment.

**Figure 5:**
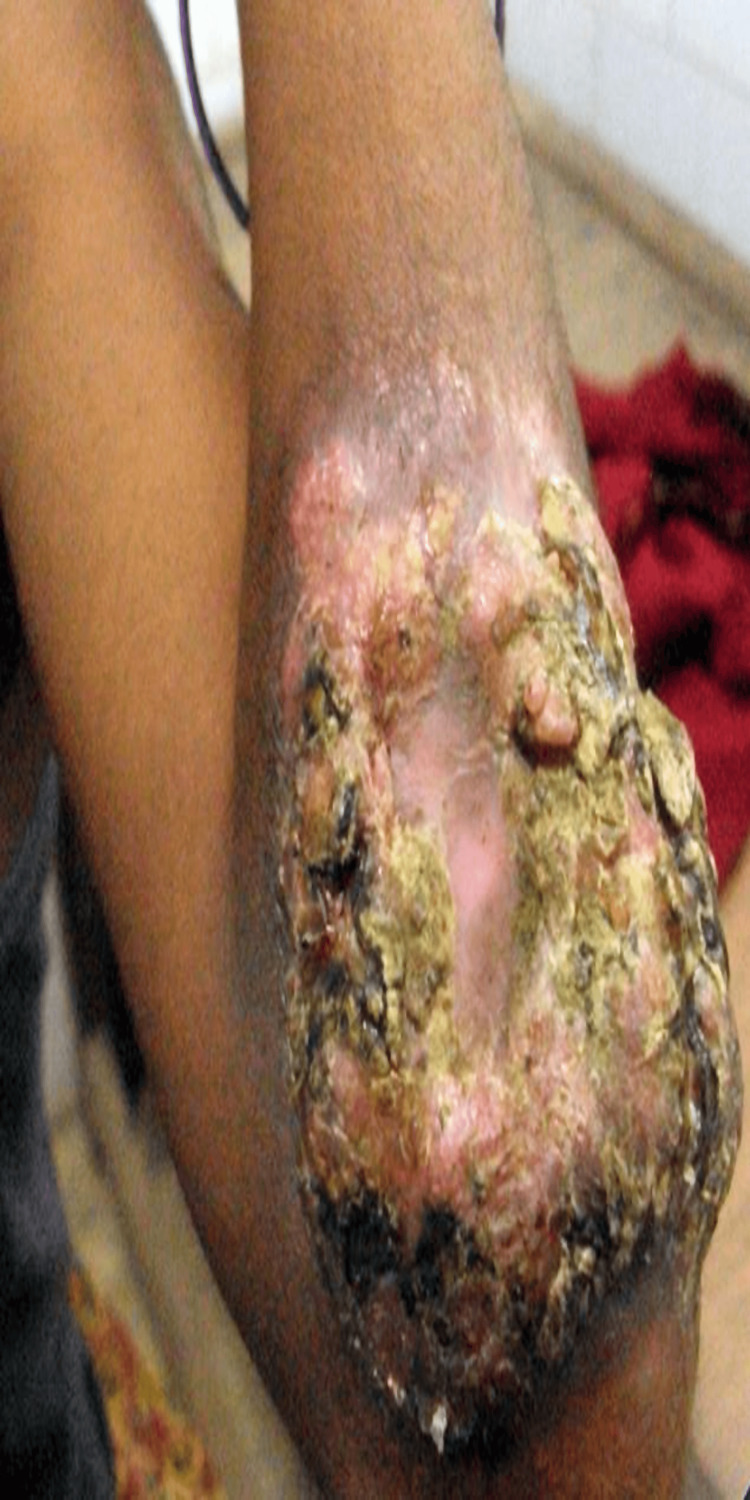
Plaque-type lesion over left elbow, before treatment.

**Figure 6:**
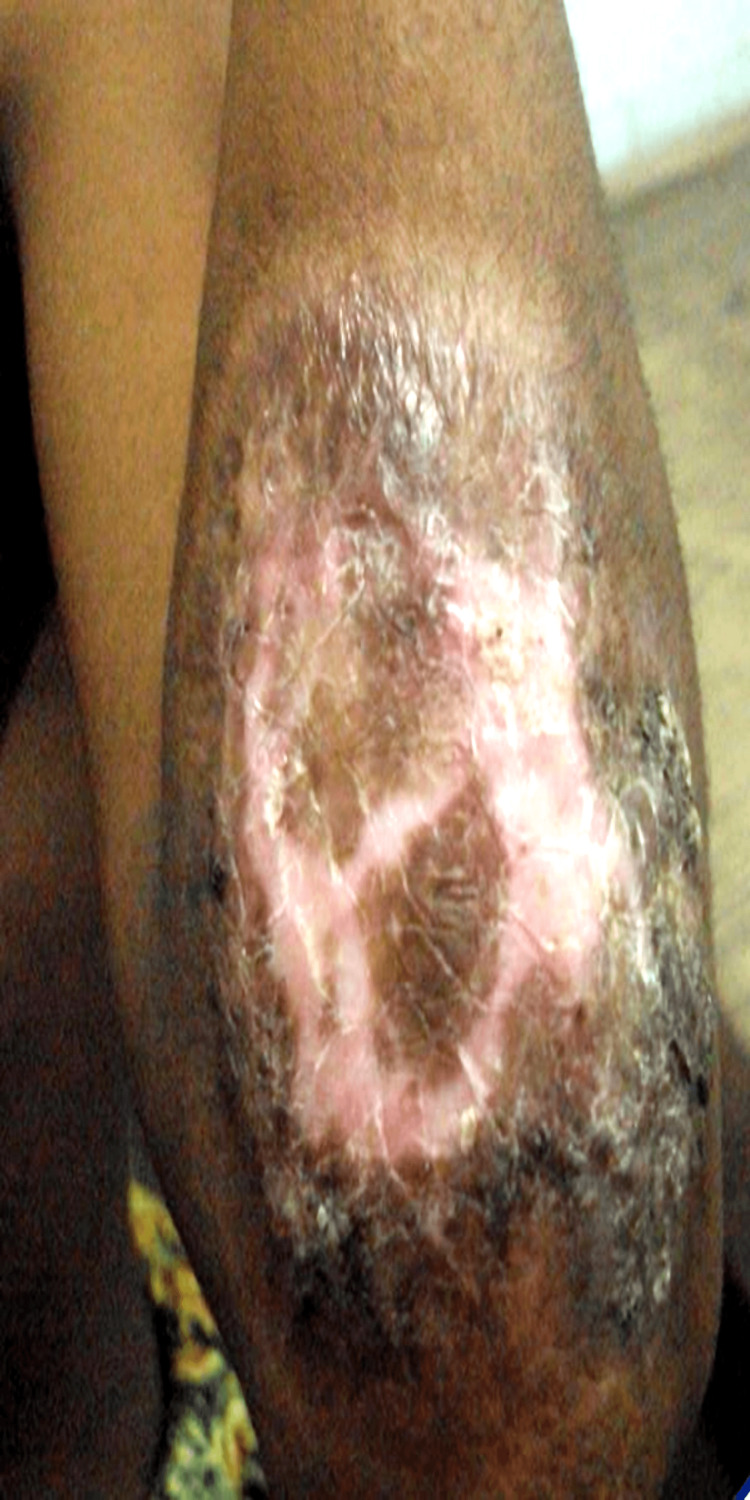
Healed lesion over elbow, after treatment.

**Figure 7:**
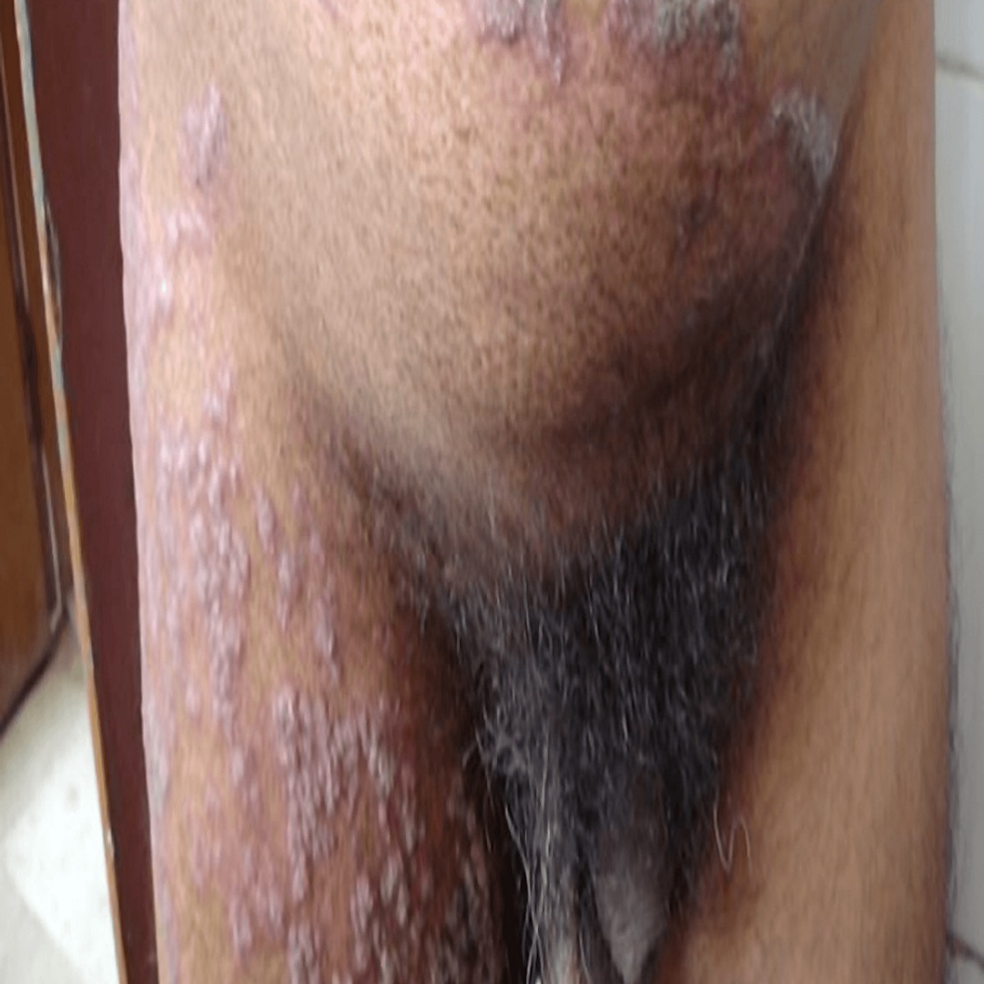
Annular plaque lesion affecting lower abdomen and upper thigh, before treatment.

**Figure 8:**
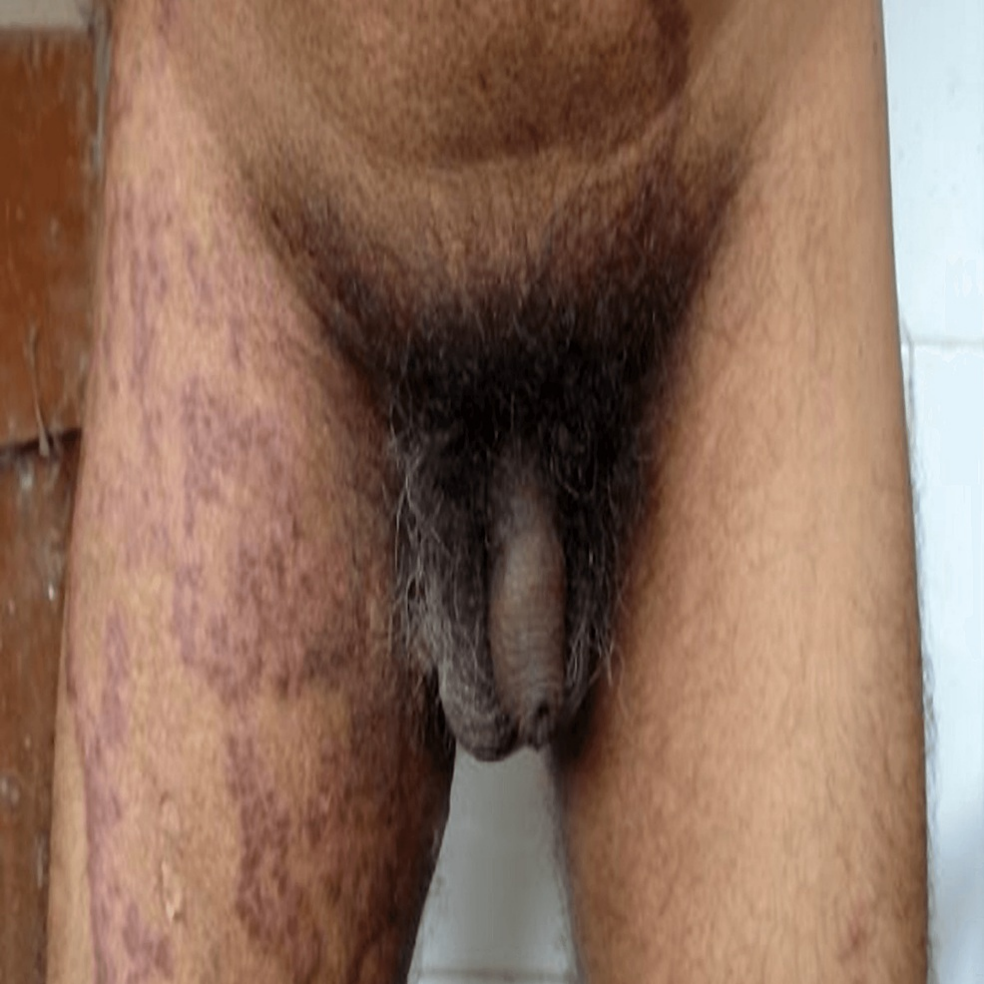
Healed thigh and lower abdomen lesion, after treatment

**Figure 9:**
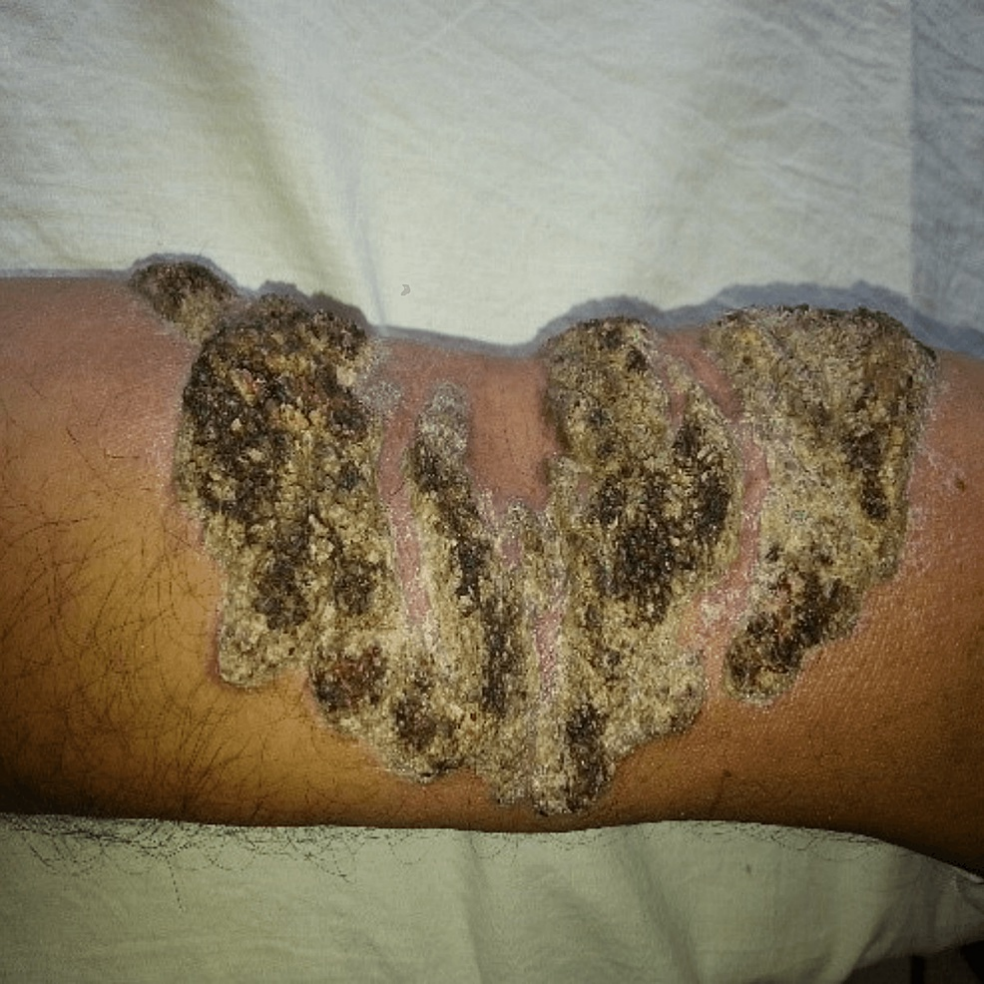
Hyperkeratotic plaque over ante-cubital fossa, before treatment.

**Figure 10:**
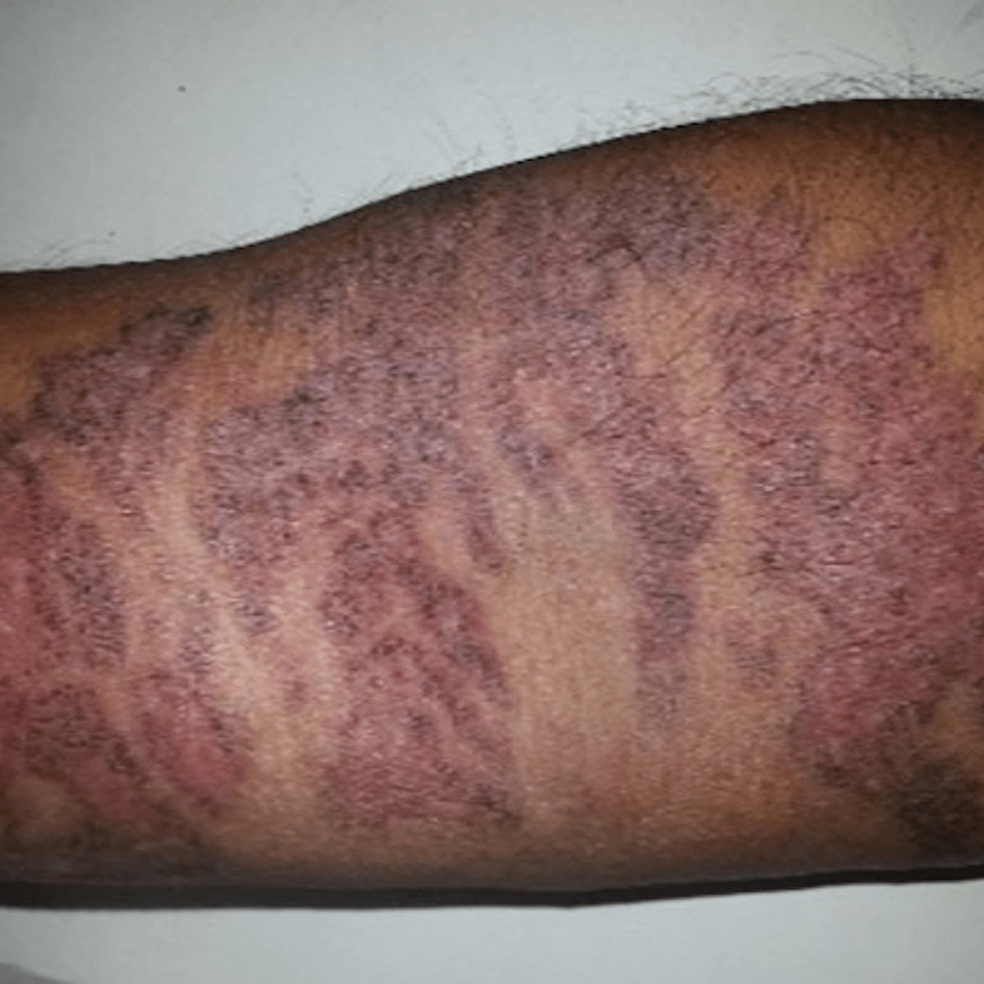
Healed lesion over antecubital fossa, after treatment

In cases where the diagnosis becomes challenging and a favorable clinical response is observed within four to six weeks, a therapeutic trial of anti-tubercular therapy may be contemplated [23]. All of our patients were histopathologically diagnosed and demonstrated satisfactory responses to anti-tubercular therapy (Figures 3-10) without any indications of drug resistance.

Within the confines of this study, our sample size was limited. To establish the validity of our findings, it is imperative to conduct additional studies involving larger cohorts from centers characterized by comparable socioeconomic, cultural, and climatic conditions. 

## Conclusions

The current study predominantly exhibited plaque type of LV with only one instance of the vegetating type. The younger age groups (5-40 years) were more susceptible compared to older individuals. Affliction primarily affected the lower limbs and buttocks in the majority of cases. Histopathological examination revealed the presence of tubercular granuloma lesions in all cases, devoid of any malignant alterations. All lesions exhibited favorable responses to conventional anti-tubercular therapy.
